# Dental effects of enzyme replacement therapy in case of childhood-type hypophosphatasia

**DOI:** 10.1186/s12903-021-01673-2

**Published:** 2021-06-27

**Authors:** Rena Okawa, Kazuma Kokomoto, Kazuhiko Nakano

**Affiliations:** Department of Pediatric Dentistry, Osaka University Graduate School of Dentistry, 1-8 Yamada-oka, Suita, Osaka 565-0871 Japan

**Keywords:** Hypophosphatasia, Enzyme replacement therapy, Hypomineralization, Dental age, Mandibular bone density

## Abstract

**Background:**

Hypophosphatasia (HPP), a skeletal disease characterized by hypomineralization of bone and teeth, is caused by an *ALPL* gene mutation that leads to low activity of the tissue non-specific alkaline phosphatase enzyme. Although enzyme replacement therapy (ERT) was recently introduced for affected patients, no known studies have been reported regarding its dental effects related to permanent teeth and jaw bones. In the present study, we examined the dental effects of ERT in a case of childhood-type hypophosphatasia, including panoramic radiography findings used to estimate the dental age of permanent teeth and mandibular bone density. Furthermore, the effects of that therapy on the periodontal condition of the patient were evaluated by comparing periodontal pocket depth before and after initiation.

**Case presentation:**

An 11-year-1-month-old boy was referred to our clinic for consultation regarding oral management. Two primary incisors had spontaneously exfoliated at 1 year 8 months old and he had been diagnosed with childhood-type HPP at the age of 2 years 2 months. Obvious symptoms were localized in the dental region at the time of diagnosis, though later extended to other parts of the body such as bone pain. ERT was started at 11 years 7 months of age, after which bone pain disappeared, and motor functions and activities of daily living improved. We estimated dental age based on tooth development stage. The age gap between chronological and dental ages was expanded before treatment, and then showed a constant decrease after ERT initiation and finally disappeared. The index for mandibular bone density (mandibular cortical width / length from mesial buccal cusp to apex of first molar) was increased after ERT initiation. Furthermore, the periodontal condition for all teeth except those exfoliated was stable after starting therapy.

**Conclusions:**

ERT resulted in improved tooth and mandibular bone mineralization, with notably good effects on teeth under formation. Acceleration of mineralization of roots associated with erupting teeth leads to stabilization of the periodontal condition. We concluded that ERT contributed to the improved dental condition seen in this patient.

**Supplementary Information:**

The online version contains supplementary material available at 10.1186/s12903-021-01673-2.

## Background

Hypophosphatasia (HPP; OMIM entry #241500) is an inherited skeletal disorder caused by a mutation of the tissue non-specific alkaline phosphatase gene, which results in a reduced level of alkaline phosphatase enzyme activity in serum [1–4].

Some characteristic symptoms in patients with HPP are bone hypomineralization and early loss of primary teeth [5–9]. HPP is classified into six types based on the time of onset and symptoms: perinatal severe (fetal–neonatal; characterized by respiratory insufficiency and hypercalcemia), prenatal benign (fetal–neonatal; characterized by prenatal skeletal manifestations that slowly resolve into a milder HPP type), infantile (before 6 months of age; rickets without elevated serum alkaline phosphatase (ALP)), childhood (6 months–less than 18 years of age; characteristics range from low bone mineral density for age with unexplained fractures to rickets and premature loss of primary teeth with intact roots), adult (after 18 years; characterized by stress fractures and pseudofractures of the lower extremities in middle age), and odonto-HPP (any age; characterized by premature exfoliation of primary teeth without skeletal manifestations) [4–9].

Enzyme replacement therapy (ERT) using human bone‐targeting recombinant alkaline phosphatase has been developed and shown to improve prognosis [10–14]. In Japan, that therapy was introduced in 2015, earlier than other countries [15, 16]. A few studies have reported regarding the dental effects of ERT [17, 18], though those were case reports with focus on only primary teeth. In the present study, we examined the dental effects of ERT in a case of childhood-type hypophosphatasia, including panoramic radiography findings to estimate dental age of permanent teeth. Furthermore, the effects of that therapy on periodontal condition were evaluated by comparing periodontal pocket depth before and after its initiation.

## Case presentation

### Clinical history

An 11-year-1-month-old Japanese boy was referred to the Pediatric Dentistry Clinic of Osaka University Dental Hospital from the Pediatric Clinic of Osaka University Medical Hospital for consultation regarding oral management. Two mandibular primary incisors had spontaneously exfoliated at 1Y8M. Thereafter, the patient had been diagnosed with childhood-type HPP at the age of 2Y2M due to a low serum alkaline phosphatase (ALP) level (66 IU/I), as well as radiological examination findings of the lower extremities and hands, which revealed rickets, a metaphyseal irregularity. The obvious symptoms were localized in the dental region at diagnosis, though they later extended to other parts of the body such as bone pain. ERT was performed by subcutaneous injections of recombinant bone-targeted ALP at 2 mg/kg three times weekly and commenced at 11Y7M.

We previously reported dental findings of this patient at the age of 11Y5M, obtained before initiating ERT therapy [19]. Briefly, an intraoral examination demonstrated that all incisors and canines, as well as the first molar had emerged into the oral cavity, each of which showed enamel hypoplasia, while all first molars were located in a prominently lower position. Since the maxillary and mandibular molars did not contact when biting, traumatic occlusion was thought to have occurred in areas near the incisors. A periodontal examination revealed deep pockets and severe mobility in the maxillary right central incisor and mandibular left central incisor regions. In an orthopantomographic examination, all permanent teeth except for the mandibular left second premolar were identified, however, permanent tooth root formation was delayed, and the mandible and maxilla bones appeared to be thin. A periapical radiographic examination showed severe absorption of the alveolar bone in the regions of the maxillary right central incisor and mandibular left central incisor.

### Clinical dental analysis before and after initiation of ERT

#### Intra-oral examination findings 2.5 years after starting ERT (14Y1M)

**Fig. 1:**
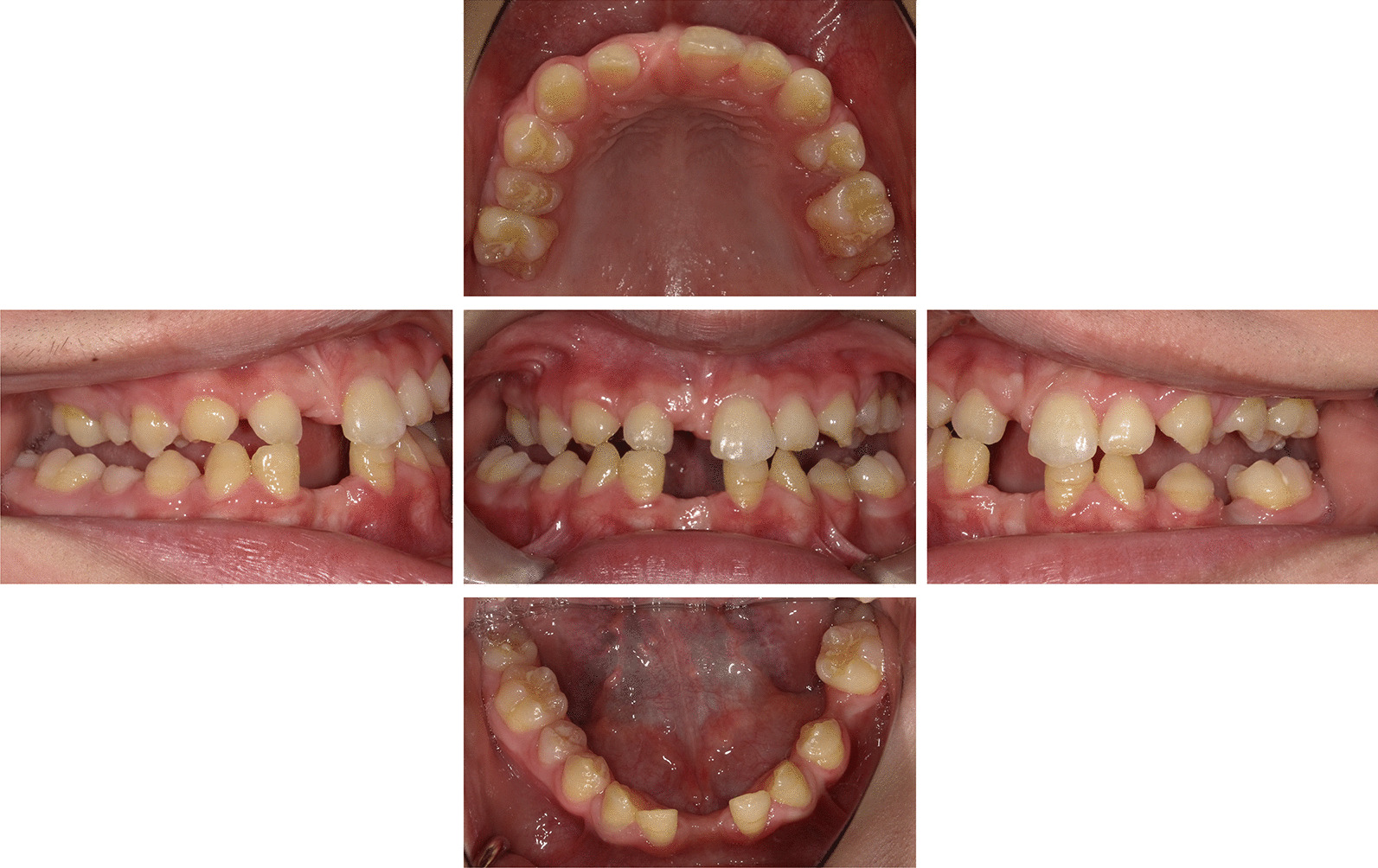
Oral photographs obtained at most recent examination (patient age 14Y9M)

The patient had been suffering from low motor function due to leg bone pain. At 6 months after initiation of ERT, he noted that the bone pain had disappeared. Thus, ERT improved motor function and activities of daily living in our patient. Following initiation of ERT, 3 permanent incisors, including the maxillary right central incisor and mandibular bilateral central incisors, were exfoliated (Fig. 1). Mobility of each of those teeth due to occlusal trauma were recognized prior to beginning therapy. In addition, the mandibular bilateral primary second molar was exfoliated, after which permanent lateral dentition excluding the maxillary and mandibular right second premolars erupted. The maxillary left second premolar did not erupt due to space loss. All permanent teeth were found to have enamel hypomineralization.

#### Periapical radiography results

**Fig. 2:**
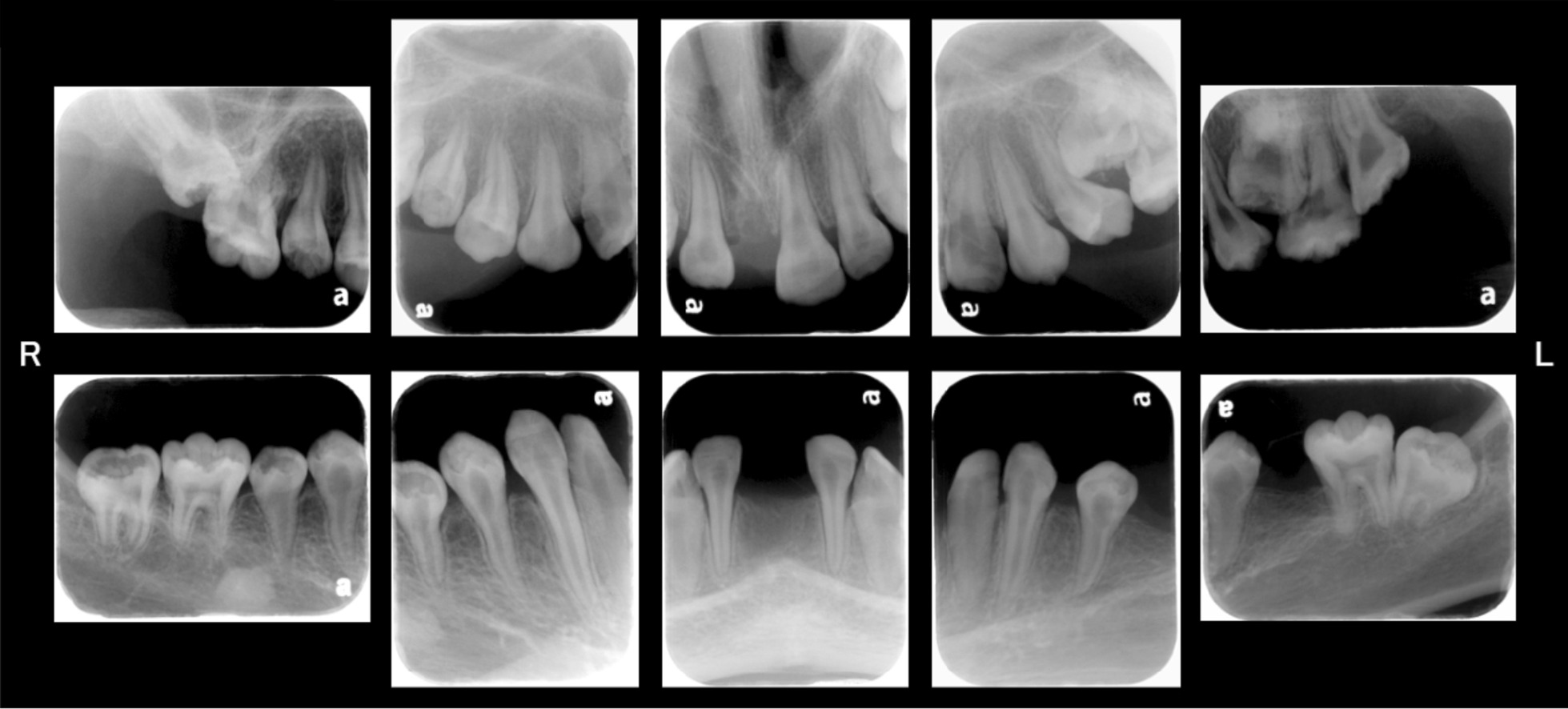
Periapical radiograph images obtained at 14Y9M of age

A periapical radiographic examination performed at 14Y9M revealed mild horizontal alveolar bone resorption around all teeth (Fig. 2), while widening of the periodontal ligament space in the anterior tooth region and disappearance of lamina dura in part of the first molar were detected. However, no widening of the periodontal ligament space was recognized around the apex of the premolar root. All tooth roots were short and thin, and pulp spaces were wide.

#### Panoramic radiography results

**Fig. 3:**
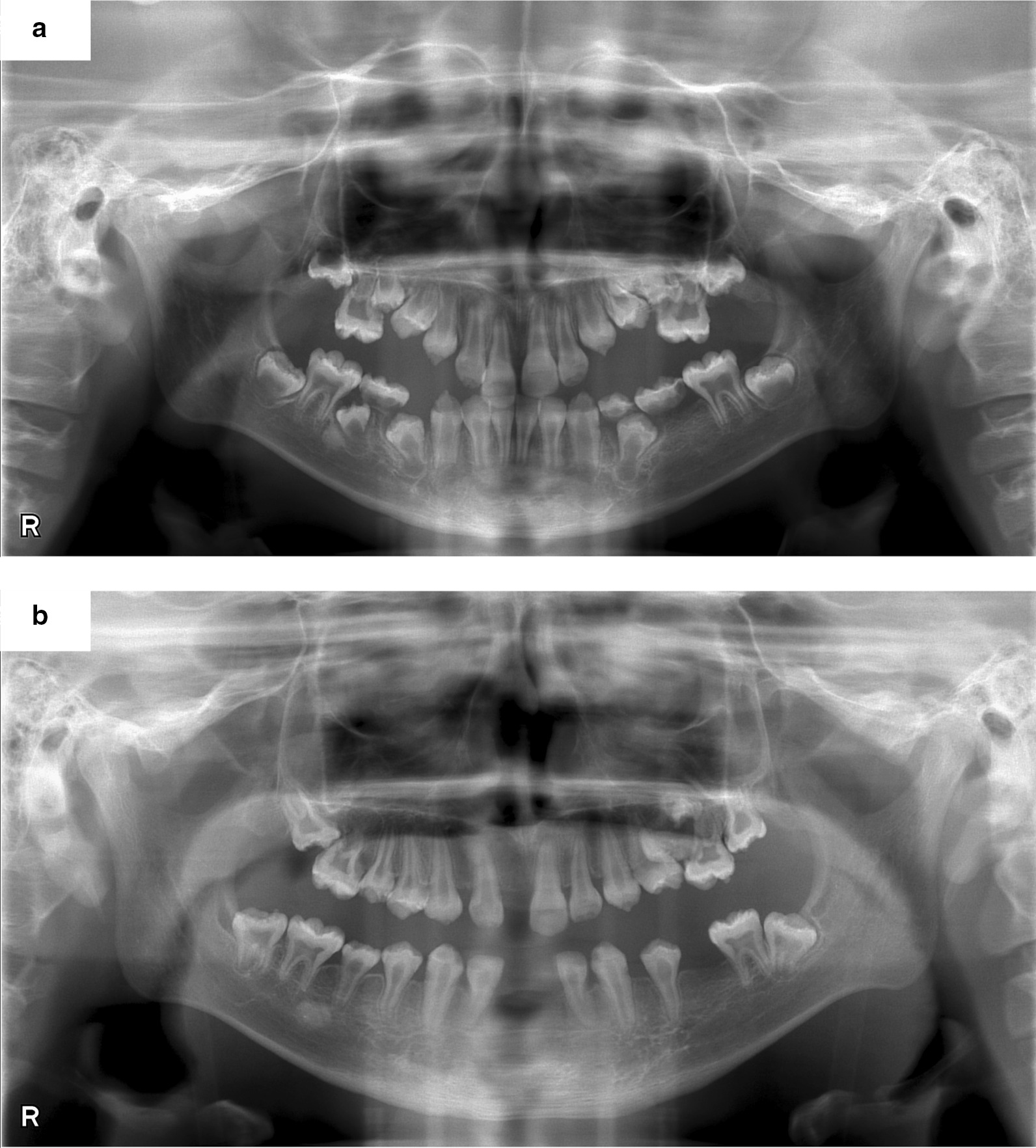
**a** Panoramic radiograph obtained at 11Y1M of age, prior to starting ERT. **b.** Panoramic radiograph obtained after starting ERT (patient age 14Y1M)

**Fig. 4:**
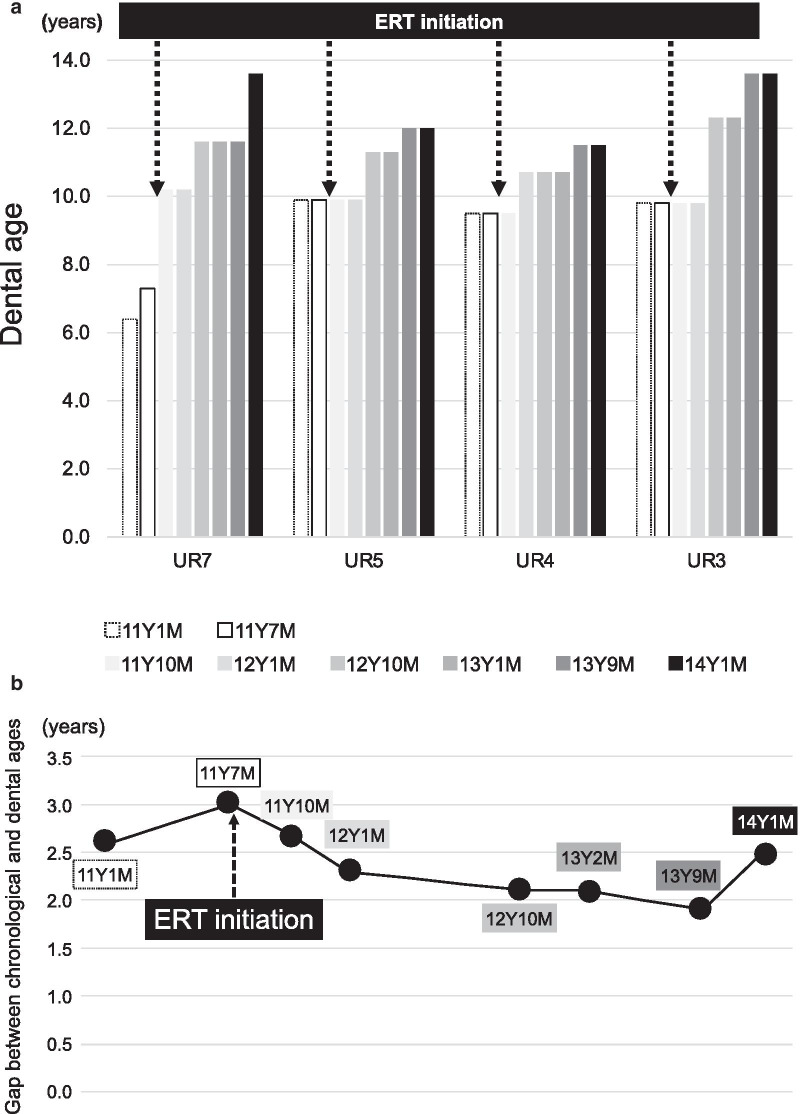
**a** Advancement of dental age. **b.** Gap between chronological and dental ages

Closing of the root apex in the incisors and first molars, and development of roots of canines and premolars were recognized as compared before initiation of ERT (Fig. 3a, b). We estimated dental age based on the development stage of permanent teeth using the method of Haavikko [20], which has been shown to be valid for application in Japanese subjects [21]. Haavikko reported age medians in years for 12 tooth formation stages for boys and girls separately, as well as for the maxilla and mandible. In this study, a single pediatric dentist assessed formation stage of all permanent teeth using panoramic radiography images. The formation stages were then converted to chronological age and the average of those chronological ages was considered to be the dental age of the patient. For the present patient, results from a total of 8 panoramic radiography sessions, 2 prior to beginning treatment at the age of 11Y1M and 11Y7M, and 6 after starting treatment at 11Y10M, 12Y1M, 12Y10M, 13Y1M, 13Y9M, and 14Y1M of age, were analyzed (Additional file 1). The canines, first and second premolars, and second molars showed prominent growth during the examination period, as those teeth were in the process of development (Fig. 4a). The dental age of the canines and premolars before beginning therapy was approximately 10 years, and that of the second molar was 7 years. However, at 2.5 years after initiation of ERT dental age was approximately 12 years old. Thus, the gap between chronological and dental age was reduced after beginning therapy (Fig. 4b).

**Fig. 5:**
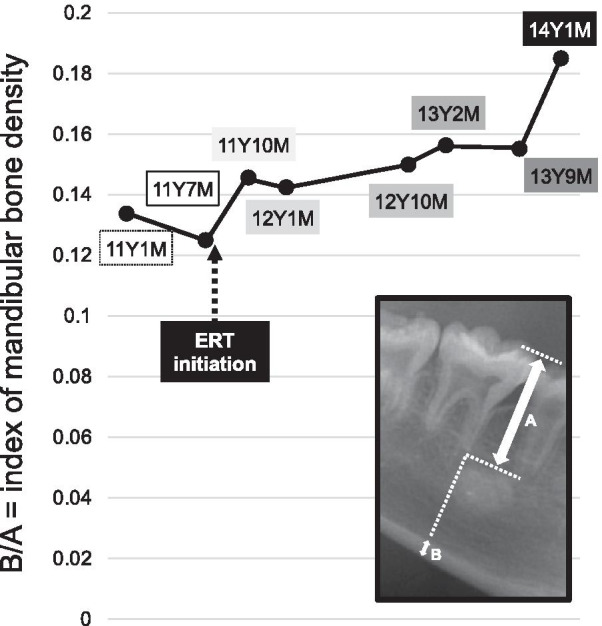
Change in mandibular bone density index. Area enclosed by square illustrates method for estimation of bone density

The results in this case showed that during the 6-month period before treatment, the age gap had expanded, while it showed a constant decrease during the 2.5 years after ERT initiation and finally disappeared. On the other hand, the central and lateral incisors, and first molars did not show significant growth during that time. Several studies have evaluated bone mineral density using mandibular cortical width shown in panoramic radiographs in children with other types of skeletal diseases or for clinical osteoporosis screening [22–25]. However, there are limitations related to differences in experience and agreement among different observers, as well as image quality and orthopantomography magnification. For the present case, we utilized a modified quantitative evaluation method for determining bone mineral density using mandibular cortical width by correcting the length of the first molar, and calculated mandibular bone density as follows: mandibular cortical width / length from mesial buccal cusp to apex of first molar. Our findings showed that the index of mandibular bone density was increased after ERT initiation (Fig. 5).

#### Periodontal condition

**Fig. 6:**
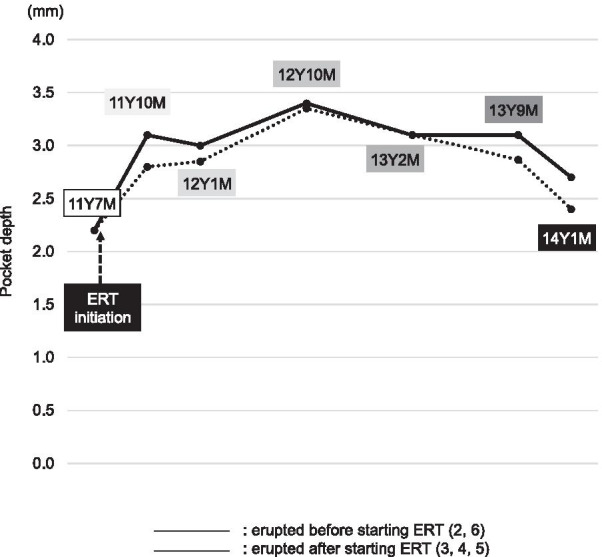
Improvements in periodontal scores

Periodontal examination findings revealed deep pockets and severe mobility in the incisor regions, while only mild gingival inflammation was noted. We determined pocket depth (6-point method) and mobility before (11Y7M) and after (11Y10M, 12Y1M, 12Y10M, 13Y1M, 13Y9M, 14Y1M) starting ERT. The average pocket depth, except for the incisors and first molars, remained at approximately 3 mm during that period (Fig. 6). No mobility of the canines and premolars that erupted after initiation ERT was noted.

## Discussion

In HPP patients, early loss of permanent teeth is less frequent as compared with primary teeth [5, 8]. As for the present case, severe periodontitis in 3 central incisors was recognized prior to initiation of ERT, then those were exfoliated after starting therapy. Disturbed cementum formation was also confirmed in both primary and permanent teeth in this case [19] with occlusal trauma causing excessive pressure without adequate molar occlusion consider to be a trigger of periodontitis. This problem highlights the importance of space maintenance and occlusal guidance for early loss of primary teeth. However, no widening of space for the periodontal ligament was recognized around the apex of the premolar root, which we speculated was an effect of ERT on periodontal tissue formation.

A previous study reported dental caries and tooth loss as treatment-emergent adverse events in HPP treated with ERT, though no dental criteria were noted regarding the diagnosis of “tooth loss” [13]. The present is the first case report that includes comparisons of dental changes before and after starting ERT. We used dental age to estimate the effects of ERT on permanent teeth. Although tooth formation in the present patient was found to be delayed before ERT initiation, root growth caught up to chronological age over time, indicating that ERT is effective for mineralization of teeth. In the jaw bone as well, the index of mandibular bone density was increased. However, use of that index is not an established method for bone density calculation [22], thus panoramic radiography performed under controlled conditions is necessary to clarify the density of mandibular bone.

All except for exfoliated teeth showed stable eruption both before and after ERT, which we considered to indicate a stable periodontal condition, especially in regard to the alveolar bone. No tooth mobility was seen after starting ERT, showing that the therapy had good effects on periodontal tissue. In the present patient, crown formation was completed before initiating ERT and enamel and dentine hypomineralization of all teeth was found. ALP plays an important role in not only bone but also tooth mineralization. Had ERT begun prior to tooth germ formation, enamel and dentin hypomineralization might have been improved.

The present findings revealed good tooth and jaw bone formation in our patient, as the effects of ERT are focused on bone hypomineralization, especially in severe type HPP cases [11]. Enzyme administration during the period of jaw growth and permanent tooth formation may improve development of the oral region. ERT was initiated only 4 years ago in Japan and dental effects of have yet to be fully elucidated. We intend to continue research with objective indicators to clarify the dental effects of ERT.

No fundamental dental treatment for HPP has been established. This is the first known case report documenting the dental effects of ERT related to permanent teeth and jaw bones in affected patients. In conclusion, the present findings indicate that initiation of ERT led to improvements in not only periodontal condition, but also tooth and bone mineralization. Collaboration between pediatricians and pediatric dentists is important for improved quality of life of HPP patients in regard to bone and dental conditions.

## Supplementary Information

**Additional file 1**. Dental age. Dental ages of incisors, first and second premolars, and second molars were evaluated from results obtained in a total of 8 panoramic radiography sessions.

## Data Availability

All data generated or analysed during this study are included in this published article and its supplementary information files.

## Abbreviations

ALP: Alkaline phosphatase
ERT: Enzyme replacement therapy
HPP: Hypophosphatasia
